# Comparative analyses of *Pleurotus pulmonarius* mitochondrial genomes reveal two major lineages of mini oyster mushroom cultivars

**DOI:** 10.1016/j.csbj.2024.01.021

**Published:** 2024-02-01

**Authors:** Yang Yu, Tianhai Liu, Yong Wang, Lixu Liu, Xiaolan He, Jianwei Li, Francis M. Martin, Weihong Peng, Hao Tan

**Affiliations:** a Sichuan Institute of Edible Fungi, Sichuan Academy of Agricultural Sciences, Chengdu 610000, China; b Sichuan Agricultural University, Chengdu 610000, China; c Sichuan Academy of Agricultural Sciences, Chengdu 610000, China; d Université de Lorraine, INRAE, UMR Interactions Arbres/Microorganismes, Centre INRAE Grand Est, Nancy, Champenoux 54280, France; e Northwest Institute of Eco-Environment and Resources, Chinese Academy of Sciences, Lanzhou 730000, China

**Keywords:** *Pleurotus pulmonarius*, Commercialized cultivars, Wild isolates, Maternal origin, Conserved PCG arrangement, *Dpo* gene, Intronic homing endonuclease

## Abstract

*Pleurotus pulmonarius*, commonly known as the mini oyster mushroom, is highly esteemed for its crisp texture and umami flavor. Limited genetic diversity among *P. pulmonarius* cultivars raises concerns regarding its sustainable industrial production. To delve into the maternal genetic diversity of the principal *P. pulmonarius* cultivars, 36 cultivars and five wild isolates were subjected to *de novo* sequencing and assembly to generate high-quality mitogenome sequences. The *P. pulmonarius* mitogenomes had lengths ranging from 69,096 to 72,905 base pairs. The mitogenome sizes of *P. pulmonarius* and those of other mushroom species in *the Pleurotus* genus showed a significant positive correlation with the counts of LAGLIDAG and GIY-YIG homing endonucleases encoded by intronic open reading frames. A comparison of gene arrangements revealed an inversion of a fragment containing *atp9-nad3-nad2* between *P. pulmonarius* and *P. ostreatus*. The mitogenomes of *P. pulmonarius* were clustered into three distinct clades, two of which were crowded with commercial cultivars. Clade I, all of which possess an inserted *dpo* gene, shared a maternal origin linked to an ancestral cultivar from Taiwan. Primers were designed to target the *dpo* gene, potentially safeguarding intellectual property rights. The wild isolates in Clade III exhibited more divergent mitogenomes, rendering them valuable for breeding.

## Highlights

• Mitogenomes were analyzed among 41 isolates of the mini oyster mushroom.
• Gene arrangement distinguished P. pulmonarius from closely related Pleurotus species.
• Mitogenomic size of Pleurotus genus correlated with homing-endonuclease-gene counts.
• P. pulmonarius cultivars had a narrow range of maternal genetic diversity.
• Primers were designed to identify Taiwan’s maternal origin by targeting the dpo gene.

## Graphical Abstract

**Figure:**
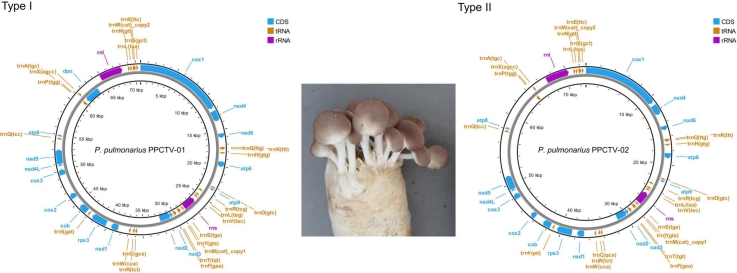

## 1 Introduction

*Pleurotus pulmonarius* (Fr.) Quel., commonly referred to as the mini oyster mushroom or lung-shaped oyster mushroom, is a member of the Pleurotaceae family within the Agaricales order of the Basidiomycota phylum. This mushroom is globally recognized and renowned for its culinary appeal. Initially labeled *Pleurotus geesteranus*, commercialized cultivars of the mini oyster mushroom were eventually reclassified as *P. pulmonarius* [1]. In terms of appearance, the basidioma of the mini oyster mushroom is smaller than that of the common oyster mushroom, *Pleurotus ostreatus*. Notably, the young basidiomata of both species exhibit similar characteristics, leading to their colloquial names.

The basidioma of *P. pulmonarius* has a distinctive crisp-tender texture and an umami flavor profile [2]. Rich in essential nutrients, such as proteins, vitamins, minerals, and essential amino acids [3], [4], this mushroom also contains a variety of bioactive compounds, including fungal polysaccharides, ergosterol, and γ-aminobutyric acid [5], [6]. Its consumption has been associated with a wide range of health benefits, including antioxidative, antidiabetic, antiaging, and immune-enhancing effects [3], [6], [7], [8]. Due to its impressive adaptability and stress resistance, *P. pulmonarius* can thrive on diverse agricultural and forestry waste materials [5]. This unique trait enables it to yield basidiomata during high-temperature seasons, thereby addressing the scarcity of common oyster mushrooms in the summer market [9]. Given its attributes, *P. pulmonarius* is anticipated to emerge as a significant alternative to the prevailing common oyster mushroom, positioning itself as a prominent edible mushroom species.

Mainland China holds the distinction of being the world’s largest producer of the mini oyster mushroom [1]. Although *P. pulmonarius* is distributed in the wild across China [1], [10], the lineage of commercialized cultivars in mainland China can be traced back to Taiwan [9]. Long-term domestication, selection, and cultivation by local mushroom growers and technicians in mainland China [9] beginning in the 1990 s has contributed to the gradual differentiation in agronomic traits between commercialized *P. pulmonarius* cultivars. Over the ensuing decades, the proliferation and diversification of these cultivars in mainland China reflect a process of mutation and evolution within a new habitat. Although certain wild *P. pulmonarius* germplasms may have contributed to the hybrid breeding of commercialized cultivars, the majority appear to have been derived from their Taiwanese ancestors. However, owing to the long-term lack of historical breeding records, the genetic background of these cultivars is poorly understood. Consequently, the genetic foundations of these cultivars remain enigmatic, a situation that is further complicated by discrepancies in nomenclature. As a result, there is a need to comprehensively survey and classify the spectrum of commercialized *P. pulmonarius* cultivars that prevail on the market. The intricacies surrounding the genetic origins and relationships of these cultivars require in-depth exploration. Shedding light on their provenance and lineage, as well as disentangling their intricate genetic networks, is a promising direction for the advancement of the breeding sector of mini oyster mushroom.

Genes encoded by mitochondrial DNA are involved in a series of physiological and biochemical processes, such as cell growth and development, stress resistance, energy metabolism, aging, and apoptosis [11], [12], [13]. The mitochondrial genome is considered the second genome of eukaryotes [14]. Mitochondrial DNA, which is maternally inherited and does not undergo recombination during sexual reproduction, is haploid, making it relatively easy to analyze. It has been utilized to resolve phylogenetic relationships among closely related species in a genus, or even between different strains of the same species [15], [16], [17]. This study aimed to use the mitochondrial genome to dissect the phylogenetic relationships of 36 commercialized cultivars of *P. pulmonarius* and five isolates collected from wild environments.

## 2 Materials and methods

### 2.1 *Pleurotus pulmonarius* isolates

Thirty-six isolates of the major commercialized *P. pulmonarius* cultivars were supplied by leading enterprises for mini oyster mushroom production, and five isolates were collected from wild forests (Table S1). Commercial cultivars and wild isolates were cultivated to produce basidiomata, and their major agronomic traits were recorded (Table S1). The substrate used for *P. pulmonarius* cultivation contained 57% (w/w) sawdust, 30% (w/w) cottonseed husk, 10% (w/w) wheat bran, 2% (w/w) CaCO_3_, and 1% (w/w) CaSO_4_. In addition, a wild isolate of *P. ostreatus* was included in the analysis for comparison. All isolates were obtained from dikaryotic cultures. All live isolates were obtained from the Germplasm Collection Bank of the Sichuan Institute of Edible Fungi (Chengdu, Sichuan Province, China).

### 2.2 DNA extraction and sequencing

Fresh basidioma tissue from the mushroom was used for total DNA extraction, including both nuclear and mitochondrial DNA. The Rapid Fungi Genomic DNA Isolation Kit (Sangon Biotech Inc., Shanghai, China) was used for DNA extraction following the procedures described in the manufacturer’s instructions. The NEBNext® Ultra™ II DNA Library Prep Kit (NEB, Beijing, China) was used to construct PE400 sequencing libraries following the manufacturer’s instructions. Sequencing was performed by Frasergen Bioinformatics Co., Ltd. (Wuhan, China) on an Illumina NovaSeq platform (Illumina, San Diego, CA, USA). Each isolate yielded 5 Gb of raw sequencing data. Sequencing adapters in the raw reads were removed using AdapterRemoval v2 [18]. Low-quality sequences were filtered using ngsShoRT [19] with default parameters.

### 2.3 Assembly of the mitochondrial genome

GetOrganelle version 1.7.7 was used to assemble the mitochondrial genome (mitogenome) from quality-controlled Illumina reads [20]. The GetOrganelle fungal database was used to identify, filter, and assemble target-associated reads using default parameters (command line: get_organelle_from_reads.py −1 forward.fq −2 reverse.fq -R 10 -k 21,45,65,85,105 -F fungus_mt -o fungus_mt_out) [21]. Forty-one *P. pulmonarius* mitogenomes and one *P. ostreatus* mitogenome were assembled into complete circular contigs as provided by GetOrganelle. The average sequencing depth for each isolate is listed in the supplementary information (Table S2). For comparison, the mitogenomes of *Pleurotus ostreatus* DSM11191 and PC15 [22], *Pleurotus citrinopileatus* CGMCC5.838 [16], *Pleurotus cornucopiae* SWS-15 [23], *Pleurotus eryngii* ATCC90797 [24], and *Pleurotus giganteus* Shenxun NO.1 [25] were downloaded from publicly accessible databases from the webpage addresses listed in the supplementary information (Table S3).

### 2.4 Mitochondrial gene annotation

The online tool MFannot (http://megasun.bch.umontreal.ca/cgi-bin/dev_mfa/mfannotInterface.pl) was used to annotate the assembled mitogenomes of *P. pulmonarius*, *P. ostreatus*, *P. citrinopileatus*, *P. cornucopiae*, *P. eryngii*, and *P. giganteus*. The mold mitochondrial genetic code was selected for running the MFannot tool, as previously described [26]. Annotation was used to identify protein-coding genes (PCGs), rRNA genes, and tRNA genes. The exon/intron boundaries of the PCGs were confirmed and manually curated, as previously described [17], [27]. All mitogenomes were manually adjusted to begin with the start codon of cox1 to facilitate comparison and alignment. Mitogenomic maps were illustrated using the CGView online tool [28].

### 2.5 Identification of repeated sequences

Repeated sequences were identified using the methods previously described by Li, Chen [16]. Briefly, intra-mitogenomic repeats were identified by BLASTn searches of the whole mitogenomic sequence against itself, at an E-value < 10^−10^. Tandem repeats were searched using the Tandem Repeats Finder, which is available online, with default parameters.

### 2.6 Calculation of AT/GC skew

AT/GC skew was used to measure the asymmetry of the base composition in the mitogenomic DNA strand using the method described by Li, Li [29]: AT skew = (A - T)/(A + T), and GC skew = (G − C)/(G + C). The AT/GC skew was calculated for each of the 41 *P. pulmonarius* mitogenomes and mitogenomes of the other species in the *Pleurotus* genus mentioned previously.

### 2.7 Verification of dpo gene presence

Three pairs of primers were designed to examine the presence of the *dpo* gene in the *P. pulmonarius* mitogenome. The primers were manually designed using Oligo 7.53 Primer Analysis Software (Molecular Biology Insights Inc., Cascade, CO, USA) to anneal the *dpo* gene template at 60–65 °C and to avoid any potential hairpin or dimer structures. To avoid false-positive results caused by nonspecific amplification, only when all three pairs of primers had positive results was the presence of the *dpo* gene.

### 2.8 Prediction of the tRNA secondary structure

tRNAscan-SE v2.0 was used to confirm the sequences of the tRNA genes and predict the secondary structures of the tRNA products [30]. The parameters of the sequence source and genetic code for tRNA isotype prediction were selected as “Other mitochondrial” and “Mold&Protozoan Mito”, respectively. The predicted structural files were visualized using Forna online software [31].

### 2.9 Analysis of genetic variants

Each conserved PCG of the 41 *P*. *pulmonarius* mitogenomes was aligned using MEGA-X. Pairwise genetic distances were calculated using MEGA-X based on the Kimura two-parameter (K2P) substitution model between each pair of the 41 *P*. *pulmonarius* mitogenomes, as described by Tan, Yu [32]. DnaSP v6.10 was used to calculate the nucleotide diversity per site (*π*), haplotype diversity (*Hd*), and average number of nucleotide differences (*k*) of the conserved PCGs and rRNA genes [33]. Evolutionary models of conserved PCGs and rRNA genes were estimated by Tajima’s *D* test using DnaSP v6.10, as described by Liu, He [34]. Nonsynonymous substitution rates (*Ka*) and synonymous substitution rates (*Ks*) of conserved PCGs were calculated using DnaSP v6.10.

Mutations of single nucleotide polymorphisms (SNPs) and insertions/deletions (indels) were analyzed by GATK version 4.17 [35] and filtered for minimum allele frequency (maf) and linkage disequilibrium (LD) as previously described by Zhang, Xu [15]. The commercial cultivar Taixiu 57 (*P. pulmonarius* isolate PPCTV-01) was used as a template for comparison. The filtered SNPs and Indels were annotated with ANNOVAR [36].

### 2.10 Phylogenetic analysis

SNPs in the mitogenomes were used to analyze the phylogenetic relationships among the *P. pulmonarius* isolates. The filtered SNPs were converted to phylip format using vcf2phylip version 2.4 [37]. A phylogenetic tree was constructed with MEGA-X [38] using the maximum likelihood method with 1000 bootstraps. The “find best DNA/Protein models (ML)” function in MEGA-X was used to select the most suitable model for constructing the maximum likelihood tree, and the general time reversible (GTR) model with uniform rates was selected for its low BIC score. The parameters of “Gaps/Missing Data Treatment” were set to “Partial deletion” with a site coverage cutoff of 95%. Wild *P. ostreatus* isolate POW-01 was used as an outgroup taxon.

### 2.11 Data accessibility

The mitogenome sequences of the isolates presented in this study were deposited in GenBank under accession numbers OR265946–OR265987.

## 3 Results

### 3.1 Mitogenome overview

**Fig. 1:**
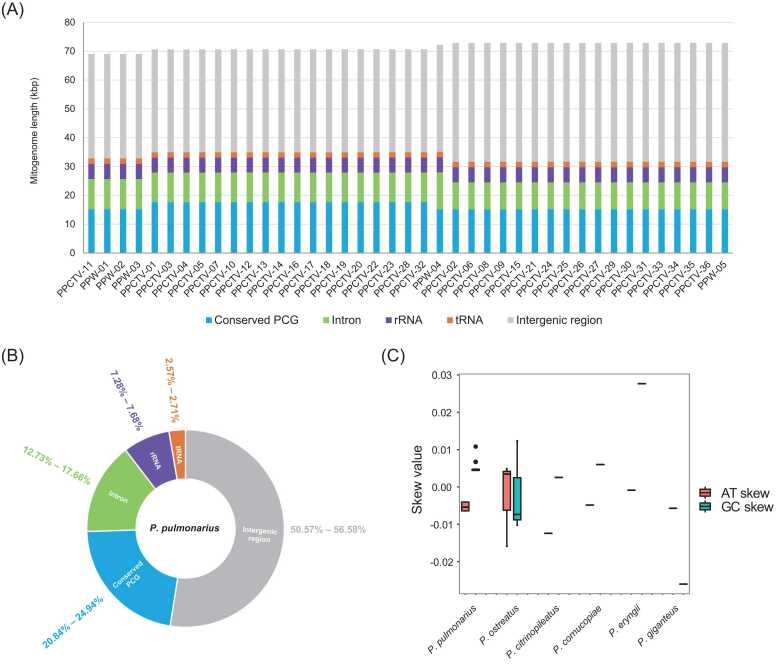
(A) Length distribution of the 41 *Pleurotus pulmonarius* mitogenomes; (B) Proportion (%) of different components in the *P. pulmonarius* mitogenomes; (C) AT/GC skew of the *P. pulmonarius* mitogenome and comparison with the other five species in the *Pleurotus* genus.

Complete circular mitogenomes were assembled from high-throughput Illumina sequencing reads of 41 *P. pulmonarius* isolates and one *P. ostreatus* isolate. The sequencing depth of these multiple mitogenomes was > 5000 × (Table S2). Among the 41 *P. pulmonarius* isolates, we identified five mitogenomes with distinct lengths. Specifically, there were 4 cases of 69,096 bp, 1 case of 69,104 bp, 18 cases of 70,674 bp, 1 case of 72,275 bp, and 18 cases of 72,905 bp (Fig. 1 **A**). Notably, the lengths of the *P. pulmonarius* mitogenomes aligned closely with those of counterparts, such as *P. ostreatus* POW-01 (71,426 bp), *P. cornucopiae* SWS-15 (72,134 bp), and *P. eryngii* ATCC90797 (72,745 bp). Comparatively, they were longer than that of *P. citrinopileatus* CGMCC5.838 (60,694 bp) but shorter than that of *P. giganteus* Shenxun NO.1 (102,950 bp).

The mitogenome of *P. pulmonarius* contained 16 conserved protein-coding genes (PCGs), accounting for 20.84–24.94% of the total assembly length. Two rRNA genes, *rnl* and *rns*, accounted for 7.28–7.68% of the mitogenome, while twenty-five tRNA genes accounted for 2.57–2.71%. The introns in the conserved PCGs accounted for 12.73–17.66% of the mitogenome, while the intergenic regions had more than half the total length (50.57–56.58%) (Fig. 1**B**).

The widely recognized commercial cultivar Taixiu 57, denoted as PPCTV-01 and introduced from Taiwan in the 1990 s, had a mitogenome length of 70,674 bp. Remarkably, this length was shared by 17 other commercial cultivars, including PPCTV-03 (marketed as Xiangmeijun), PPCTV-04 (Penghui), and PPCTV-20 (Xuedong). For the most part, these cultivars have been developed through the efforts of mushroom growers. In contrast, a separate group comprising 17 cultivars and the wild isolate PPW-05 exhibited mitogenomes with a length of 72,905 bp. Hence, these two distinct mitogenome lengths, 70,674 bp and 72,905 bp, characterized the genetic makeup of commercialized *P. pulmonarius* cultivars.

Three distinct wild isolates, PPW-01, PPW-02, and PPW-03, sourced from a national nature reserve in Ganzi Prefecture, exhibited a mitogenome measuring either 69,096 or 69,104 bp. This 8-bp variation resulted from the presence of a small insertion fragment, GCGTAGCA, within the intergenic region flanked by the tRNA genes *trnY(gta)* and *trnT(tgt)*. The commercialized cultivar Yongtai No.3, designated PPCTV-11, showed a mitogenome length of 69,096 bp. Another wild isolate identified as PPW-04, collected from a national nature reserve in Aba Prefecture, displayed a considerably longer mitogenome, measuring 72,275 bp, distinguishing it from the remaining wild isolates.

The 41 *P. pulmonarius* mitogenomes had an average AT skew of − 5.221 ± 1.141 × 10^−3^ and a GT skew of 4.945 ± 1.149 × 10^−3^ (Fig. 1**C**). All *P. pulmonarius* mitogenomes had a negative AT skew and positive GC skew. This indicates that *P. pulmonarius* mitogenome DNA had a slightly higher content of T than A, and a slightly higher content of G than C. Specifically, the 18 cultivars with a 70,674-bp mitogenome had identical sequences, with an AT skew of − 6.418 × 10^−3^ and a GC skew of 4.425 × 10^−3^. The 17 cultivars with a 72,905-bp mitogenome had an AT skew of − 4.036 × 10^−3^ and a GC skew of 4.739 × 10^−3^, with two exceptions. PPCTV-25 and PPCTV-33 both had a T→G mutation at the 63,581-bp position within the intergenic region between the *atp8* and *trnA(tgc)* genes, leading to a change in AT and GC skew to − 4.018 × 10^−3^ and 4.794 × 10^−3^, respectively.

### 3.2 Gene arrangement

**Fig. 2:**
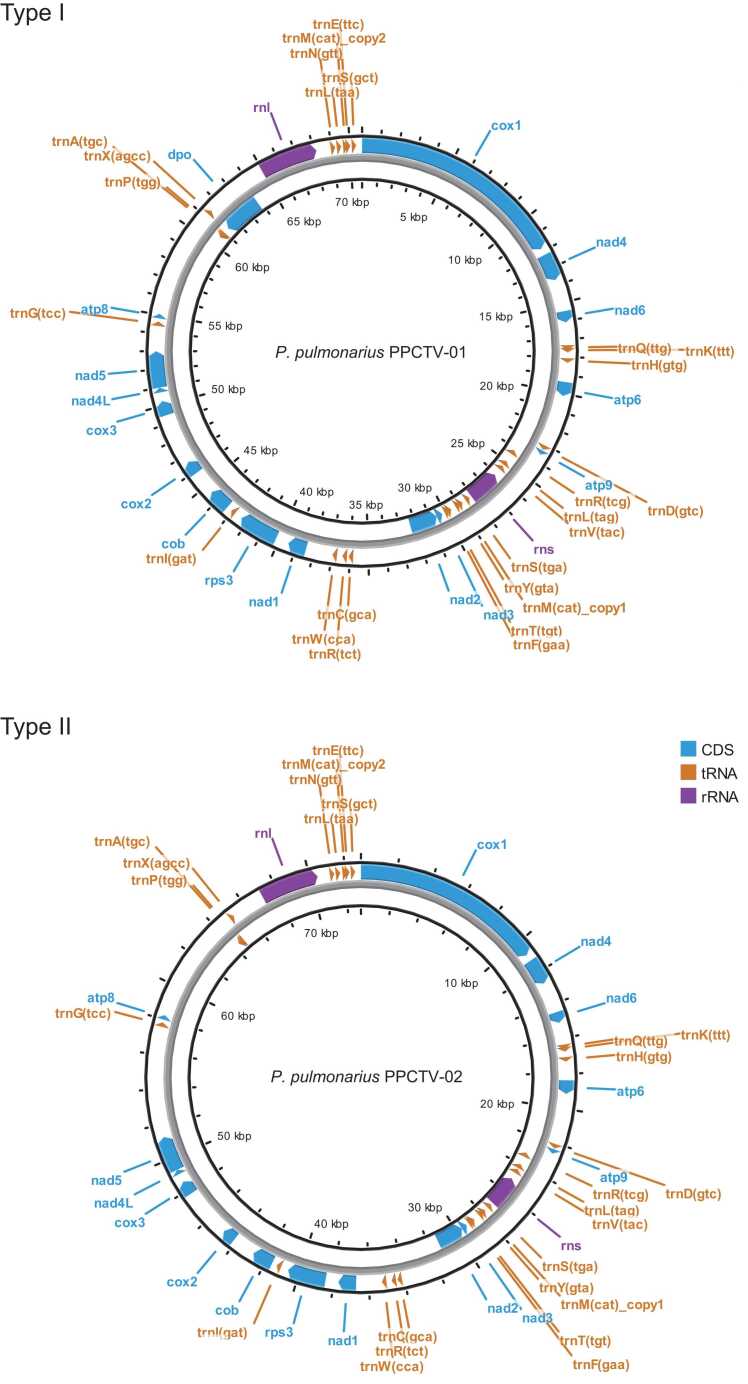
Circular maps of *P. pulmonarius* mitogenomes. Type I and Type II were defined by the presence of the *dpo* gene.

The organization of the 16 conserved PCGs within the mitogenomes of *P. pulmonarius* followed a sequential arrangement: *cox1-nad4-nad6-atp6-atp9-nad3-nad2-nad1-rps3-cob-cox2-cox3-nad4L-nad5-atp8-(dpo)* (Fig. 2). The genes *cox1, cox2*, and *cox3* encode subunits of cytochrome c oxidase; *cob* is responsible for cytochrome b synthesis; *atp6*, *atp8*, and *atp9* encode subunits of ATP synthase; *nad1*, *nad2*, *nad3*, *nad4*, *nad4L*, *nad5*, and *nad6* encode subunits of the NADH dehydrogenase complex III in the electron transport chain; *rps3* encodes the ribosomal protein S3; and *dpo* encodes a DNA polymerase.

Notably, the *cox1* gene displayed an unusual GTG start codon instead of a typical ATG codon. Within the *P. pulmonarius* mitogenomes, the presence of the *dpo* gene was confined to the 70,674-bp variants, whereas it was conspicuously absent in mitogenomes measuring 69,096, 69,104, 72,275, and 72,905 bp. This differentiation, based on the presence or absence of the *dpo* gene, delineated two primary types of *P. pulmonarius* mitogenomes. The 70,674-bp mitogenome cultivars constituted Type I, whereas Type II included 72,905-bp mitogenome cultivars and 69,096, 69,106, and 72,275-bp mitogenome wild isolates (Fig. 2). This classification facilitated the design of three pairs of primers, determining whether a given cultivar possessed a Type I mitogenome (Table S4).

Three of the conserved PCGs, *nad3*, *nad2*, and *dpo*, were located in the reverse strand, whereas all other conserved PCGs were in the forward strand. Of the 25 tRNA genes, 16 were located on the forward strand, while the remaining nine were located on the reverse strand. Furthermore, *rnl*, the rRNA gene that encodes the large subunit of the ribosome, was located in the forward strand, while the small subunit gene *rns* was in the reverse strand.

**Fig. 3:**
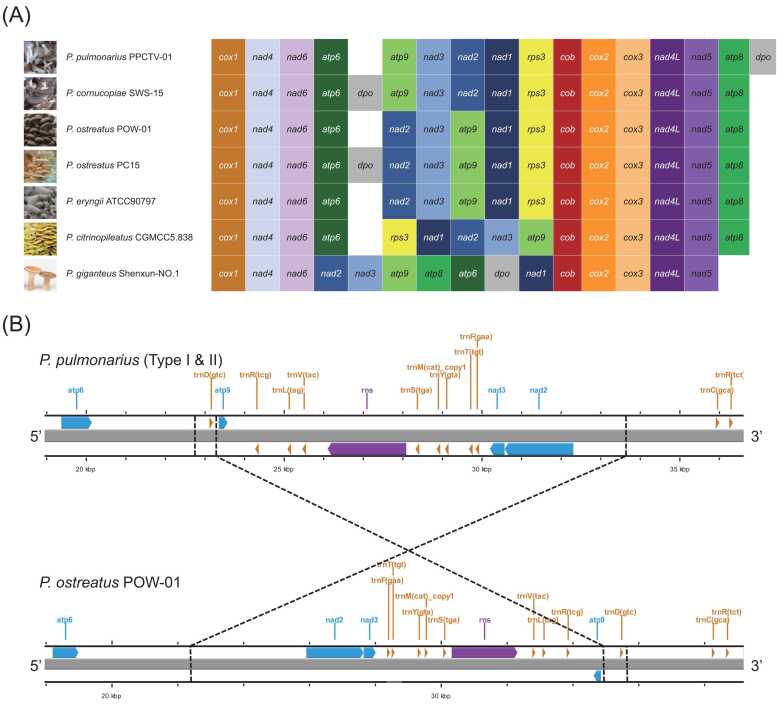
(A) Gene arrangement of conserved PCGs compared among six *Pleurotus* mushrooms: *P. pulmonarius*, *P. cornucopiae*, *P. ostreatus*, *P. eryngii*, *P. citrinopileatus*, and *P. giganteus*; (B) Schematic diagram showing the inversion of the *atp9*-*nad3*-*nad2* fragment between *P. pulmonarius* and *P. ostreatus*.

The organization of conserved PCGs was subjected to comparative analysis between *P. pulmonarius* and several other widely consumed mushroom species within the *Pleurotus* genus (Fig. 3**A**). The findings demonstrated a nearly identical arrangement of conserved PCGs between *P. pulmonarius* and *P. cornucopiae*, with the sole divergence being the insertion of the *dpo* gene near the 3′-end of *P. pulmonarius* mitogenome Type I, as opposed to its placement between the *atp6* and *atp9* genes in *P. cornucopiae*. In the mitogenomes of *P. pulmonarius* and *P. ostreatus*, a noteworthy inversion of the *atp9-nad3-nad2* fragment was observed. This inversion encompassed all constituent genes within the fragment, including the conserved PCGs, tRNA genes, and rRNA gene *rns*. Specifically, this fragment appeared to be excised from the 3′-end of *trnD(gtc)* in *P. pulmonarius*, reversed, and subsequently integrated into the 5′-end of *trnD(gtc)* in *P. ostreatus* (Fig. 3**B**). Analogous inversions of the *atp9-nad3-nad2* fragment were also observed in the mitogenomes of *P. eryngii* and *P. giganteus*, albeit with variations, such as the migration of the *atp6* and *atp8* genes in *P. giganteus*. Furthermore, the *P. citrinopileatus* mitogenome exhibited inversion of a longer *atp9-nad3-nad2-nad1-rps3* fragment (Table S5).

### 3.3 Intron gain/loss in cox1

**Fig. 4:**
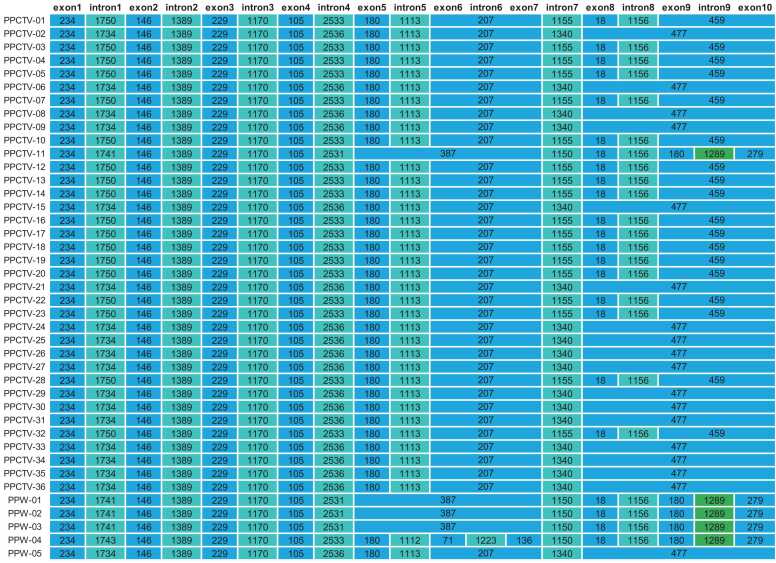
Arrangement of exons and introns in *cox1* among the 41 *Pleurotus pulmonarius* isolates, showing intron gain/loss and exon amalgamation. Exons are colored in light blue, group IA introns are green, and group IB introns are teal.

In the *P. pulmonarius* mitogenome, PCG *cox1* was the only gene containing introns (Fig. 4). The *cox1* gene was composed of a maximum of 10 exons and nine introns, a configuration identified in the PPW-04 isolate. Among these, exon 1, exon 2, exon 3, exon 4, intron 1, intron 2, intron 3, and intron 7 maintained a consistent structure across the 41 mitogenomes, while the other exons and introns displayed varying degrees of variability. Specifically, exon 1, exon 2, exon 3, exon 4, intron 2, and intron 3 maintained identical lengths across all *P. pulmonarius* isolates. However, intron 7 was the most variable in terms of length.

The nine introns all contained intronic open reading frames (ORFs) encoding homing endonucleases (HEs). Introns 1–8 were all group IB introns encoding LAGLIDADG HEs, while intron 9 was the only group IA intron encoding a GIY-YIG HE. Intron 9 was only present in the four wild isolates (PPW-01 to PPW-04) and one commercial cultivar, PPCTV-11. These HEs granted the introns the potential to reshuffle the hosting PCG. Variations in the number of exons and introns are the result of intron gain or loss events. As expected, the loss of an intron resulted in the fusion of adjacent exons, leading to alterations in the number of exons and introns. In the case of PPW-04, the gain of intron 6 (1223 bp) resulted in the splitting of 71-bp exon 6 and 136-bp exon 7. Conversely, the loss of intron 6 resulted in the amalgamation of exons 6 and 7 into an elongated exon of 207 bp. This 207-bp exon was consistently found in most *P. pulmonarius* isolates. Furthermore, the absence of intron 5 (approximately 1113 bp) led to further amalgamation of the 207-bp exon and the 180-bp exon 5. This merged exon, totaling 387 bp, was observed in wild isolates PPW-01, PPW-02, and PPW-03, as well as in cultivar PPCTV-11 (Yongchuan NO.3). Notably, although PPCTV-11 possessed identical counts of introns in *cox1* as the other cultivars with 70,674-bp mitogenomes, the configurations of their introns were quite different. PPCTV-11 possessed the 1289-bp intron 9 belonging to group IA and lost the 1113-bp intron 5 of group IB, while the other cultivars possessed intron 5 and lost intron 9 (Fig. 4). The loss of intron 9 caused the fusion of exons 9 and 10 in PPCTV-01 combined with the absence of intron 8 led to a 477-bp exon fusion in PPCTV-02.

### 3.4 tRNA genes

Of the 25 tRNA genes identified, 24 were responsible for encoding tRNAs that transfer amino acid residues during peptide synthesis, while *trnX(agcc)* seemed to be a pseudogene (Table 1). Across all 41 *P. pulmonarius* isolates, each tRNA gene had identical sequences. Notably, tRNAs dedicated to the transfer of arginine, leucine, and serine are governed by two distinct tRNA genes, each corresponding to different codons. In contrast, the transfer of methionine was governed by two copies of the *trnM(cat)* gene, slightly differing in sequence while utilizing a CAU codon. The other tRNAs were characterized by a single tRNA gene. This observation could potentially provide valuable insights into codon usage preferences in the mitochondria of *P. pulmonarius*.

**Table 1:** Codon usage of tRNA genes in the *Pleurotus pulmonarius* mitogenome.

**Target amino acid**	**tRNA gene**	**Codon used by the tRNA**	**Codons not used by the tRNA**
Glutamine	*trnQ(ttg)*	CAA	CAG
Lysine	*trnK(ttt)*	AAA	AAG
Histidine	*trnH(gtg)*	CAC	CAU
Aspartate	*trnD(gtc)*	GAC	GAU
Arginine	*trnR(tcg)*, *trnR(tct)*	CGA, AGA	CGU, CGC, CGG, AGG
Leucine	*trnL(tag)*, *trnL(taa)*	CUA, UUA	UUG, CUU, CUC, CUG
Valine	*trnV(tac)*	GUA	GUU, GUC, GUG
Serine	*trnS(tga)*, *trnS(gct)*	UCA, AGC	UCU, UCC, UCG, AGU
Methionine	*trnM(cat) (two copies)*	AUG	AUA
Tyrosine	*trnY(gta)*	UAC	UAU
Threonine	*trnT(tgt)*	ACA	ACU, ACC, ACG
Phenylalanine	*trnF(gaa)*	UUC	UUU
Cysteine	*trnC(gca)*	UGC	UGU
Arginine	*trnR(tct)*, *trnR(tcg)*	AGA, CGA	CGU, CGC, CGG, AGG
Tryptophan	*trnW(cca)*	UGG	UGA
Isoleucine	*trnI(gat)*	AUC	AUU
Alanine	*trnA(tgc)*	GCA	GCU, GCC, GCG
Proline	*trnP(tgg)*	CCA	CCU, CCC, CCG
Asparagine	*trnN(gtt)*	AAC	AAU, AAA, AAG
Glutamate	*trnE(ttc)*	GAA	GAG

**Fig. 5:**
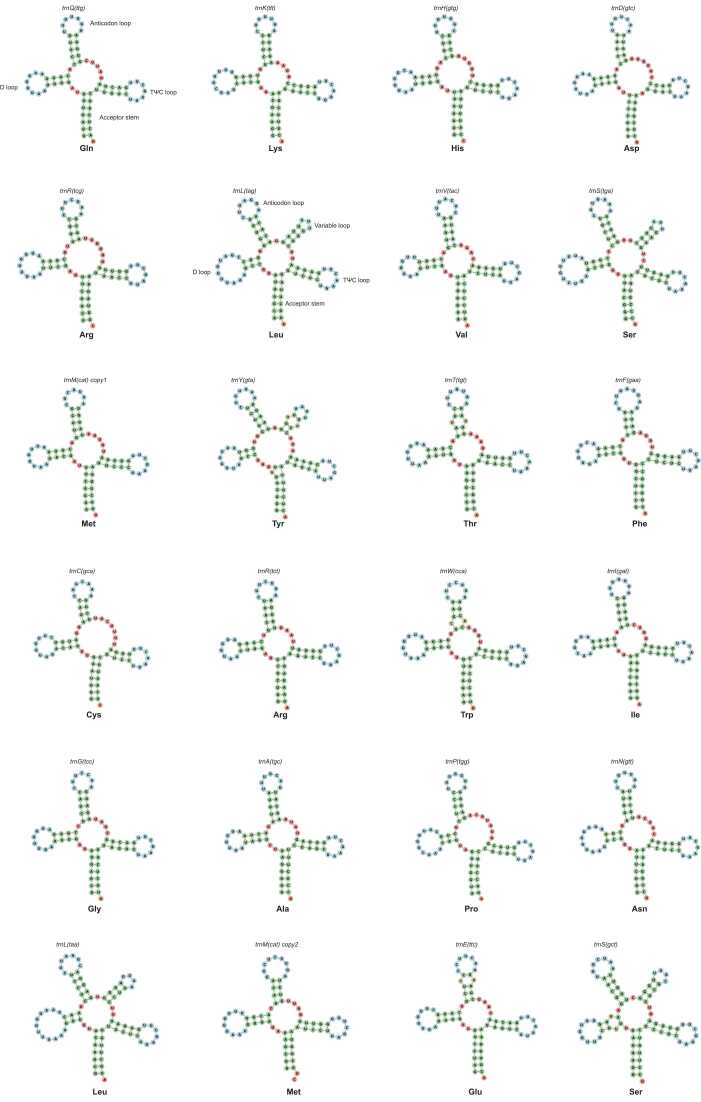
Predicted secondary structures of tRNA products encoded by tRNA genes.

In terms of their predicted secondary structures, certain tRNAs, such as leucine, serine, and tyrosine, exhibited notably elongated variable loops, endowing them with a shape reminiscent of a starfish, as opposed to the conventional cloverleaf structure (Fig. 5). In contrast, tRNAs associated with other amino acids maintain a more typical cloverleaf configuration.

### 3.5 Polymorphisms of conserved PCGs and rRNA genes

**Fig. 6:**
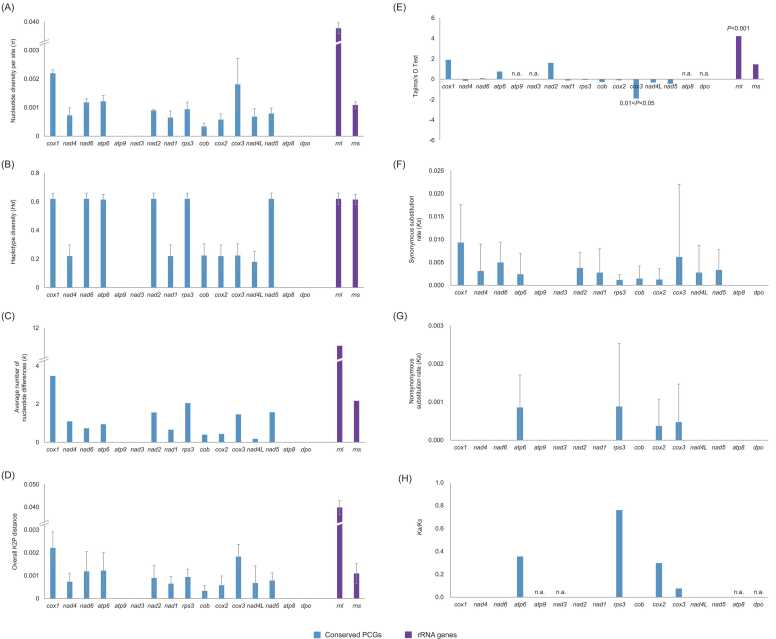
Polymorphisms of conserved PCGs and rRNA genes in 41 *Pleurotus pulmonarius* mitogenomes. Conserved PCGs arranged along the X-axis were in accordance with their order in the mitogenome. n.a. means that the value could not be calculated because there were no polymorphisms in this gene or the denominator was zero. (A) Nucleotide diversity per site of conserved PCGs and rRNA genes; (B) Haplotype diversity of conserved PCGs and rRNA genes; (C) Average number of nucleotide differences of conserved PCGs and rRNA genes; (D) Overall K2P distances of conserved PCGs and rRNA genes; (E) Tajima’s D-Test for conserved PCGs and rRNA genes, with *P-values* of significance labeled; (F) Synonymous substitution rate (*Ka*) of conserved PCGs; (G) Nonsynonymous substitution rate (*Ks*) of conserved PCGs; (H) *Ka/Ks* of conserved PCGs.

Variations in conserved PCGs and rRNA genes were assessed using multiple parameters. Among the 16 conserved PCGs, 12 exhibited nucleotide diversity per site ranging from 2.2 ± 0.1 × 10^−3^ to 3.4 ± 1.2 × 10^−4^. Notably, *cox1* showed the highest degree of nucleotide diversity (Fig. 6**A**), aligning with the intricacies observed in the intron gain/loss within *cox1* (Fig. 4). Conversely, genes such as *atp8*, *atp9*, *nad3*, and *dpo* displayed no diversity, indicating identical sequences across the 41 *P. pulmonarius* isolates. This observation was substantiated by metrics such as haplotype diversity, average number of nucleotide differences, and overall K2P distances (Fig. 6**B–D**). In terms of rRNA-coding genes, the *rns* gene exhibited a nucleotide diversity of 1.1 ± 0.1 × 10^−3^, on par with the conserved PCGs. However, the *rnl* gene displayed higher diversity (3.7 ± 0.2 × 10^−2^) compared to the conserved PCGs (Fig. 6**A**). This trend was consistent with the average number of nucleotide differences and the overall K2P distances (Fig. 6**C, D**).

Tajima’s D test revealed noteworthy insights into the specific genes. For instance, *cox3* displayed a D value of − 1.89 (0.01 < P < 0.05) (Fig. 6**E**), suggesting an excess of low-frequency polymorphisms compared to expectations, which could point to directional selection or a potential expansion in population size. Conversely, *rnl* exhibited a D value of 4.22 (P < 0.001), implying that this gene may have undergone balancing selection or experienced a decline in population size. Notably, the D values for other genes were not statistically significant (all P > 0.05), which was consistent with the neutral theory model.

When examining the nucleotide substitution rates of the conserved PCGs, the four completely conserved genes (*atp8*, *atp9*, *nad3*, and *dpo*) showed no nucleotide substitutions (Fig. 6**F, G**); *atp8*, *atp9*, *cob*, *cox1*, *dpo*, and all *nad* genes had synonymous substitutions but no non-synonymous substitutions (Fig. 6**G**). This resulted in *Ka*/*Ks* values of zero for these genes. The remaining four genes, *atp6*, *cox2*, *cox3*, and *rps3*, were more variable. The *Ka*/*Ks* values for the four conserved PCGs were all < 1 (Fig. 6**H**), indicating purifying selection.

### 3.6 Phylogenetic relationships

**Fig. 7:**
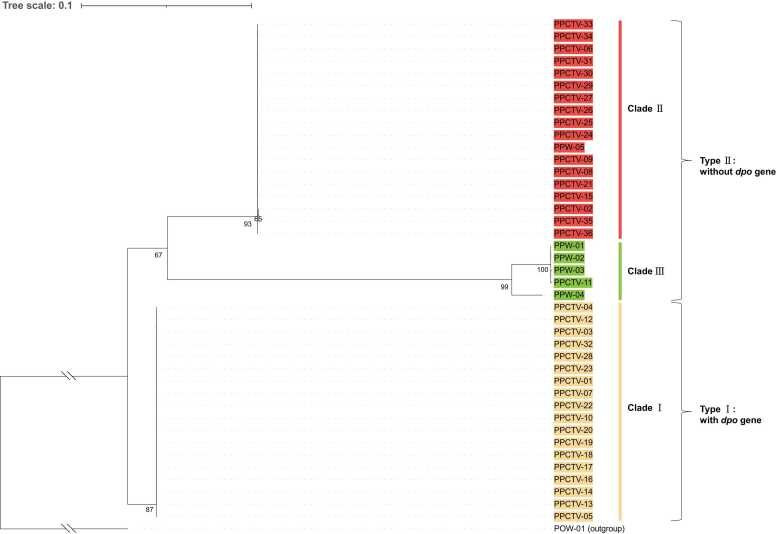
A maximum-likelihood phylogenetic tree constructed using the SNPs of the 41 *P. pulmonarius* mitogenomes, with *P. ostreatus* as an outgroup taxon. The tree is drawn to scale, with branch length measured in the frequency of substitutions per site. Bootstrap percentages higher than 50% are labeled at the nodes.

A maximum likelihood phylogenetic tree was constructed using the SNPs found in the 41 *P. pulmonarius* mitogenomes, revealing a tripartite division among them (Fig. 7). The 41 *P. pulmonarius* mitogenomes were grouped into three distinct clades. Clade I was characterized by consolidation in an individual branch. Clades II and III coalesced into the other branch. Notably, Clade I encompassed all commercialized cultivars possessing a mitogenomic length of 70,674 bp, including the PPCTV-01 and Taixiu 57 cultivars. These shared a common *dpo* gene, classifying them according to the Type I mitogenome classification (Fig. 2). In contrast, Clades II and III fell into the Type II mitogenome category (Fig. 2), featuring all 17 commercialized cultivars with 72,905-bp mitogenomes and a solitary wild isolate. In Clades I and II, uniformity in the mitogenomes reflected a shared maternal origin among the cultivars. In comparison, the mitogenomes within Clade III exhibited greater divergence. Although PPW-04, isolated near a nature reserve in Jiuzhaigou, Sichuan Province, China, showed substantial differences in mitogenome length (72,275 bp) from PPW-01, PPW-02, PPW-03, and PPCTV-11 (69,096–69,104 bp), it still aligned with Clade III rather than with Clade II because of its relatively similar length. Notably, the maternal origin of commercialized cultivar Yongchuan No.3 (PPCTV-11) was shared with wild isolates PPW-01, PPW-02, and PPW-03, indicating the involvement of wild germplasm resources in the breeding history of PPCTV-11. In combination with the different configuration of introns in *cox1* between PPCTV-11 and the cultivars with 70,674-bp mitogenomes in Clade I (Fig. 4), the results indicate that PPCTV-11 indeed had a distant relationship of maternal origin with the Clade I cultivars, despite the mitogenome length coincidence.

## 4 Discussion

Among the *Pleurotus* genus, *P. pulmonarius* (mini oyster mushroom), *P. cornucopiae* (Ji-Gu mushroom), and *P. ostreatus* (oyster mushroom) exhibit remarkably similar shapes and colors, making visual differentiation challenging. To address the needs of breeding institutions, industries, and commercial markets dealing with *Pleurotus* mushrooms, a rapid and accurate method for identifying species, both in terms of wild germplasm resources and cultivated varieties, is crucial. Conventionally, the similarity of rDNA internal transcribed spacer (ITS) sequences has been harnessed for fungal species identification. However, reliance on fixed similarity thresholds [39], such as the 99.6% threshold, may be arbitrary and liable to variations across different taxa [40], [41]. Furthermore, intragenomic variability in nuclear ribosomal markers has complicated the use of ITS for fungal species delimitation and identification [42]. Another approach involves employing phylogenetic analysis based on multiple *loci* [43], although the effectiveness of this method can be influenced by the choice of sequence alignment algorithms and tree construction techniques. Alternatively, the mitogenome is a useful tool for rapid and accurate identification of mushrooms with similar shapes or for distinguishing between diverse cultivars/strains of the same species. This mitogenome study included an analysis of 41 *P. pulmonarius* isolates, including prominent cultivars commonly employed by mushroom growers, alongside a subset of wild isolates. The mutation rate in mitochondrial DNA is usually lower than in nuclear genomes [44], [45], while exceptional examples can be observed in a few fungal taxa, such as species in the *Rhynchosporium* genus [46]. Species of *Agaricomycete* had different nucleotide substitution rates between mitochondrial and nuclear genomes [47]. Whether the *Pleurotus* genus, as well as the other common mushroom species of *Agaricales*, have a lower or higher mutation rate in their mitochondrial DNA than their nuclear genomes deserves further investigation. Nucleotide substitution rates are related to the fungal groups of Agaricomycetes [47]. A broad vertical span in the boxplots of the substitution rates of a few PCGs were observed in this study, as well as among the other species in *Agaricales* [48]. This provides some indications that nucleotide substitution frequencies of mitochondrial DNA vary between different clades of a phylogeny, such as *Agaricales*, although systematic comparisons of nucleotide substitution frequencies between different clades should be conducted to confirm this hypothesis.

In this study, different PCG gene arrangements were observed between the sequenced mitogenomes of *P. pulmonarius*, *P. cornucopiae*, and *P. ostreatus* (Fig. 3**A**). Specifically, an inverted *nad2-nad3-atp9* fragment was observed in *P. ostreatus*, distinguishing it from the other two species. Four genes, *atp8*, *atp9*, *nad3*, and *dpo*, were completely conserved in the *P. pulmonarius* population. Meanwhile, *P. cornucopiae* stood out because of the insertion of *dpo* between *atp6* and *atp9*. Another differentiating feature emerged from the loss of intron1 in *cox1*, leading to the merger of the 234-bp exon 1 and 146-bp exon 2 into an extended exon of 380 bp in *P. cornucopiae.* Similarly, the loss of intron 8 resulted in the fusion of the 18-bp exon 8 and the 180-bp exon 9 into a 198-bp exon (Fig. S1), providing further demarcation between *P. cornucopiae* and *P. pulmonarius*. As a result, it is suggested that the arrangement of mitochondrial conserved PCGs and introns in *cox1* may serve as a valuable tool for distinguishing between these three closely related *Pleurotus* species.

The inferred evolutionary trajectory of five *Pleurotus* species (*P. pulmonarius, P. cornucopiae, P. ostreatus, P. eryngii*, and *P. citrinopileatus*) was delineated based on the gene arrangement of conserved PCGs. The ancestors of these *Pleurotus* species might have a gene order similar to that found in *P. ostreatus* and *P. eryngii,* i.e*., cox1-nad4-nad6-atp6-nad2-nad3-atp9-nad1-rps3-cob-cox2-cox3-nad4L-nad5-atp8*. The subsequent inversion of the *nad2-nad3-atp9* fragment heralded the emergence of common ancestors of *P. pulmonarius* and *P. cornucopiae*. Notably, gene rearrangements have also been observed in *P. citrinopileatus* [16]. In comparison, the gene arrangement in *P. giganteus* deviated significantly from that in the other five *Pleurotus* species. This divergence corresponded well with the distinct trumpet-like morphology of the *P. giganteus* basidioma, setting it apart from the scallop shell-like shape of the other five *Pleurotus* species in their natural habitat [49], [50]. These gene reshufflings are unlikely to be attributed to any repeated sequence flanking the inversed or migrated fragments.

The AT/GC skew of mitochondrial DNA presents another potentially valuable feature for elucidating the relationships between distinct species. Notably, commercialized cultivars and wild isolates of *P. pulmonarius* exhibited a negative AT skew and a positive GC skew in their mitogenomes, which is a typical feature of the leading strand during genomic DNA duplication [51]. This means that the inferred forward strand of the mitochondrial DNA is in line with the leading strand during duplication of *P. pulmonarius* mitochondrion. This trend was similarly observed in the mitogenomes of *P. cornucopiae*, *P. citrinopileatus*, and *P. eryngii* (Fig. 1**C**). In contrast, *P. giganteus* displayed negative values for both AT and GC skew, with the GC skew being even more negatively inclined than the AT skew. This distinctive AT/GC skew pattern of *P. giganteus* is likely related to its inferred distant relationship with other *Pleurotus* species, as indicated by the gene arrangement of the conserved PCGs in their mitogenomes. The wild *P. ostreatus* isolate POW-01, isolated from China, shared a negative AT skew and a positive GC skew, similar to those of *P. pulmonarius*. Conversely, European *P. ostreatus* isolates PC15 and DSM11191 exhibited a positive AT skew and a negative GC skew. This observation suggests that the AT/GC skew may exhibit variability across distinct *P. ostreatus* isolates, potentially correlating with their geographic distribution.

**Fig. 8:**
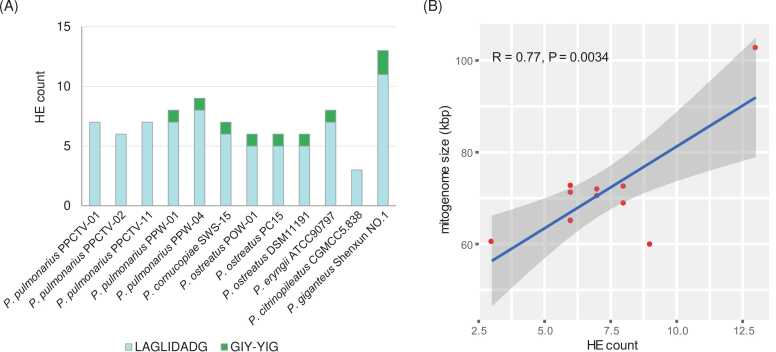
(A) Counts of intronic homing nuclease (HE) genes of LAGLIDADG and GIY-YIG in the mitogenomes of widely consumed mushroom species in the *Pleurotus* genus; (B) Correlation between intronic HE-gene counts and mitogenome size.

The mitogenomes of the *P. pulmonarius* isolates examined in this study were approximately 70 kb in size, displaying slight disparities from the wild *P. pulmonarius* isolate [52]. Mitogenome size can be affected by the number of introns, duplication of repeated elements, and new genes introduced by horizontal transfer [53], [54]. The *P. pulmonarius* mitogenomes determined in this study contained six to nine counts of LAGLIDADG or GIY-YIG HE genes, more than that of *P. citrinopileatus* and less than that of *P. giganteus* (Fig. 8**A**). *Pleurotus giganteus* had two extra HE genes (one LAGLIDADG, one GIY-YIG) in the introns of the *cob* gene, while the other *Pleurotus* species possessed only the HE genes in the introns of *cox1*. The mitogenome size of the *P. pulmonarius* isolates, together with the other species in the *Pleurotus* genus, showed a positive correlation with the counts of intronic HE genes presented in the mitogenomes (R=0.77, *P* < 0.01) (Fig. 8**B**). This is in line with previous reports that the great diversity in fungal mitogenome size was widely impacted by introns and intronic HEs [55]. This reinforced our supposition that intronic HEs might be involved in the expansion and shrinking of mitogenome size during the evolution of the *Pleurotus* genus.

Notably, owing to the inclusion of the *dpo* and *rps3* genes, *P. pulmonarius* presents the highest number of conserved PCGs (16) among Basidiomycota fungi [56]. The insertion of the *dpo* gene into the mitogenome has been observed in other edible mushrooms, such as *A. bisporus* [15] and *Agrocybe aegerita* [17]. The *dpo* gene, which encodes a DNA-directed DNA polymerase, is believed to originate from mitochondrial plasmids and can reflect the domestication history of its host [15]. Both the specific distribution patterns of the *dpo* gene and the related genetic changes in the mitogenome induced by *dpo* insertion could contribute to successful domestication of *A. bisporus* by humans [15]. However, the biological effects of *dpo* insertion on the *P. pulmonarius* mitogenomes are unclear, as there is still a lack of evidence that the *dpo* gene is actually expressed at the RNA or protein level. Further studies are necessary to examine the presence of transcriptional and/or translation products of the mitochondrial *dpo* gene in *P. pulmonarius*. The commercial *P. pulmonarius* cultivars in Clade I of the phylogenetic tree had a *dpo* gene inserted between the *trnX(agcc)* and *rnl* genes, while Clade II had no *dpo* gene. The insertion of *dpo* causes a long fragment inversion in *A. bisporus* [15], but it does not alter the order of gene arrangement in *P. pulmonarius*. In this study, primer pairs amplifying the *dpo* gene (Table S4) were designed as an easy approach to genotype *P. pulmonarius* cultivars available on the market. This marker can further identify the potential relevance of maternal origin to the Taixiu series from Taiwan. This could be a useful method for protecting intellectual property rights. Similarly, 17 cultivars with a 72,905 bp mitogenome seemed to be derived from the same ancestor, although some point mutations had taken place in PPCTV-25 and PPCTV-33.

Within the tRNA gene complement of *P. pulmonarius* mitogenomes, representation was present for all 20 amino acid residues relevant to peptide biosynthesis. This feature is typical in the majority of edible fungal species reported to date, although certain taxa, such as truffles, might lack specific tRNAs involved in transferring certain amino acid residues [57]. Among the predicted secondary structures of tRNA products encoded by the *P. pulmonarius* mitogenome, five possess long variable loops and therefore belong to the type II tRNA [58]: *trnL(tag)* and *trnL(taa)* for transferring leucine, *trnS(tga)* and trnS(gct) for transferring serine, and *trnY(gta)* for transferring tyrosine. Structural analysis revealed that the rigidity of a long variable loop in type II tRNA can lower translational processivity on the ribosome, thereby hindering the efficiency of protein synthesis [59]. Breeding new cultivars with enhanced capability of mushroom protein synthesis might be attempted via genomic editing to remove long variable loops from the tRNA products of these mitochondrial tRNA genes or to decrease the rigidity of the variable loops. The tRNA gene responsible for transferring tryptophan (*trnW*) employs diverse anticodons across species. *trnW* in the *P. pulmonarius* mitogenome uses UGG as an anticodon, which aligns with genera such as *Tuber*, *Pisolithus*, and *Trametes* [51], [60], [61]. In contrast, other fungal taxa, including *Morchella*, *Trichoderma*, *Flammulina*, *Stachybotrys*, and *Memnoniella,* utilize UGA instead [32], [62], [63], [64]. The mitochondrial *trnX* is reported to transcribe as an intermediate tRNA-like product possessing an 8-bp anticodon loop sequence but fails to further proceed as a mature tRNA product with 3′-CCA_OH_ modification [65]. This means that *trnX* appeared to be a pesudogene not participating in transferring amino acid residues during protein translation.

Approximately three decades ago, the introduction of *P. pulmonarius* cultivars from Taiwan into mainland China marked the beginning of the genetic exchange within the populations of this species. Mitochondria are inherited vertically; as such, the mitogenomes of *P. pulmonarius* cultivars could offer insights into potential maternal genetic connections with their initial Taiwanese ancestors. The current landscape of the prevalent *P. pulmonarius* cultivars is characterized by a significant degree of ambiguity, with indications that these cultivars might share origins with their Taiwanese predecessors. Our research findings indicate that the primary *P. pulmonarius* cultivars exhibit a limited range of maternal ancestries, with almost half of the cultivars (Clade I) able to be traced back to maternal roots in Taiwan. Clades I and II of *P. pulmonarius* cultivars seem to stem from ancestral sources, with mitogenomes measuring 70,674 and 72,905 bp, respectively. Cultivars with a mitogenome size of 70,674 bp were likely to share maternal origins with Taixiu 57, a well-established *P. pulmonarius* cultivar. Given the longstanding presence of Taixiu 57 as a classic cultivar in Taiwan, it is likely that certain commercialized cultivars with a mitogenome size of 70,674 bp may have evolved from the hybrid descendants of Taixiu 57 or its closely related ancestors. This could also be a result of mutations occurring within the lineage of Taixiu 57. However, wild Clade III isolates obtained from natural environments have the potential to serve as promising commercialized cultivars or foundational materials for breeding programs. This indicates that harnessing the potential of wild germplasm resources of *P. pulmonarius* could contribute to broadening the genetic diversity of commercial cultivars. In doing so, the cultivation landscape could be enriched and fortified with novel characteristics to enhance the resilience and adaptability of this important mushroom species.

The issue of naming confusion presents an additional challenge that could disrupt the market for mini oyster mushrooms. Our study demonstrated the effectiveness of comparative mitogenomics in differentiating cultivars with closely resembling names. A pertinent example is the distinction between Xiuzhen-1 (PPCTV-18) and Xiuzhen 1 (PPCTV-09). Despite their similar nomenclature, these two cultivars exhibited clear differences in their mitogenomes. Specifically, Xiuzhen-1, denoted as PPCTV-18, possesses a mitogenome size of 70,674 bp, whereas Xiuzhen 1, designated as PPCTV-09 and lacking a hyphen between "Xiuzhen" and "1,” shows a mitogenome size of 72,905 bp. This disparity in mitogenome sizes underscores the value of utilizing comparative mitogenomics to accurately distinguish and categorize cultivars that might otherwise be easily confounded by naming similarities.

## CRediT authorship contribution statement

YY: investigation, data curation, software, writing of original draft, and visualization. TL: resources, method, and investigation. YW, LL, XH, and JL: resources. FMM: conceptualization, review and editing, validation. WP: resources, validation, and funding acquisition. HT: conceptualization, investigation, supervision, writing of original draft, review and editing, and funding acquisition.

## Declaration of Competing Interest

The authors have no conflicts of interest to declare.
