# Supplementary material for: Comparative analyses of Pleurotus pulmonarius mitochondrial genomes reveal two major lineages of mini oyster mushroom cultivars
Source: Comput Struct Biotechnol J. 2024 Feb 1;23:905–17. doi: 10.1016/j.csbj.2024.01.021 (PMC10869244; doi:10.1016/j.csbj.2024.01.021)
Supplement: Supplementary file 1 — Supplementary material [file mmc1.pdf]

<table>
<tr><th></th><th>exon1</th><th>intron1</th><th>exon2</th><th>intron2</th><th>exon3</th><th>intron3</th><th>exon4</th><th>intron4</th><th>exon5</th><th>intron5</th><th>exon6</th><th>intron6</th><th>exon7</th><th>intron7</th><th>exon8</th><th>intron8</th><th>exon9</th><th>intron9</th><th>exon10</th></tr>
<tr><td>P. pulmonarius PPCTV-01</td><td>234</td><td>1750</td><td>146</td><td>1389</td><td>229</td><td>1170</td><td>105</td><td>2533</td><td>180</td><td>1113</td><td colspan="3">207</td><td>1155</td><td>18</td><td>1156</td><td colspan="3">459</td></tr>
<tr><td>P. pulmonarius PPCTV-02</td><td>234</td><td>1734</td><td>146</td><td>1389</td><td>229</td><td>1170</td><td>105</td><td>2536</td><td>180</td><td>1113</td><td colspan="3">207</td><td>1340</td><td colspan="5">477</td></tr>
<tr><td>P. pulmonarius PPCTV-11</td><td>234</td><td>1741</td><td>146</td><td>1389</td><td>229</td><td>1170</td><td>105</td><td>2531</td><td colspan="5">387</td><td>1150</td><td>18</td><td>1156</td><td>180</td><td>1289</td><td>279</td></tr>
<tr><td>P. pulmonarius PPW-01</td><td>234</td><td>1741</td><td>146</td><td>1389</td><td>229</td><td>1170</td><td>105</td><td>2531</td><td colspan="5">387</td><td>1150</td><td>18</td><td>1156</td><td>180</td><td>1289</td><td>279</td></tr>
<tr><td>P. pulmonarius PPW-04</td><td>234</td><td>1743</td><td>146</td><td>1389</td><td>229</td><td>1170</td><td>105</td><td>2533</td><td>180</td><td>1112</td><td>71</td><td>1223</td><td>136</td><td>1150</td><td>18</td><td>1156</td><td>180</td><td>1289</td><td>279</td></tr>
<tr><td>P. cornucopiae SWS-15</td><td colspan="3">380</td><td>1456</td><td>229</td><td>1170</td><td>105</td><td>2536</td><td>180</td><td>1110</td><td>71</td><td>1215</td><td>136</td><td>1347</td><td colspan="3">198</td><td>1287</td><td>279</td></tr>
<tr><td>P. ostreatus POW-01</td><td>162</td><td>1733</td><td colspan="5">480</td><td>2541</td><td>180</td><td>1112</td><td>71</td><td>1209</td><td colspan="3">154</td><td>1156</td><td>180</td><td>1287</td><td>279</td></tr>
<tr><td>P. ostreatus PC15</td><td>234</td><td>1742</td><td>146</td><td>1388</td><td colspan="7">585</td><td>1233</td><td>136</td><td>1150</td><td>18</td><td>1156</td><td>180</td><td>1283</td><td>279</td></tr>
<tr><td>P. ostreatus DSM11191</td><td>234</td><td>1742</td><td>146</td><td>1388</td><td colspan="7">585</td><td>1226</td><td>136</td><td>1335</td><td>18</td><td>1156</td><td>180</td><td>1283</td><td>279</td></tr>
</table>

Fig. S1 Comparisons of exon and intron arrangements among isolates of *Pleurotus pulmonarius*, *P. cornucopiae*, and *P. ostreatus*.
